# Metabotropic Glutamate Receptors Protect Oligodendrocytes from Acute Ischemia in the Mouse Optic Nerve

**DOI:** 10.1007/s11064-017-2220-1

**Published:** 2017-04-01

**Authors:** Arthur M. Butt, Ilaria Vanzulli, Maria Papanikolaou, Irene Chacon De La Rocha, Virginia E. Hawkins

**Affiliations:** 1 Institute of Biomedical and Biomolecular Sciences, School of Pharmacy and Biomedical Sciences, University of Portsmouth, Portsmouth, UK; 2 Department of Physiology and Neurobiology, University of Connecticut, Storrs, CT 06269 USA

**Keywords:** Oligodendrocyte, Ischemia, Hypoxia, White matter, Optic nerve, Glutamate, Metabotropic glutamate receptor

## Abstract

Studies by Bruce Ransom and colleagues have made a major contribution to show that white matter is susceptible to ischemia/hypoxia. White matter contains axons and the glia that support them, notably myelinating oligodendrocytes, which are highly vulnerable to ischemic-hypoxic damage. Previous studies have shown that metabotropic GluRs (mGluRs) are cytoprotective for oligodendrocyte precursor cells and immature oligodendrocytes, but their potential role in adult white matter was unresolved. Here, we report that group 1 mGluR1/5 and group 2 mGluR3 subunits are expressed in optic nerves from mice aged postnatal day (P)8–12 and P30–35. We demonstrate that activation of group 1 mGluR protects oligodendrocytes against oxygen-glucose deprivation (OGD) in developing and young adult optic nerves. In contrast, group 2 mGluR are shown to be protective for oligodendrocytes against OGD in postnatal but not young adult optic nerves. The cytoprotective effect of group 1 mGluR requires activation of PKC, whilst group 2 mGluR are dependent on negatively regulating adenylyl cyclase and cAMP. Our results identify a role for mGluR in limiting injury of oligodendrocytes in developing and young adult white matter, which may be useful for protecting oligodendrocytes in neuropathologies involving excitoxicity and ischemia/hypoxia.

## Introduction

Bruce Ransom has been at the forefront of white matter function in health and disease for many decades, for example as reviewed by Ransom et al. [1]. These early studies were amongst the first to demonstrate the importance of glutamate in mediating white matter damage and the protective effects of blocking ionotropic NMDA-type glutamate receptors [2]. Bruce Ransom and colleagues made important advances using the optic nerve as an archetypal CNS white matter tract and oxygen-glucose deprivation as a model for ischemia, recently providing the first direct correlation of excitoxic white matter injury in ischemia with glutamate release [3]. Moreover, Ransom and colleagues have provided evidence that CNS white matter is intrinsically more vulnerable to ischemic injury in older animals [4]. In addition to mediating axonal damage in white matter ischemia, glutamate acting via ionotropic AMPA- and NMDA-type glutamate receptors also causes damage to oligodendrocytes and myelin [5]. Indeed, glutamate-mediated excitoxicity and ischemia are features of multiple neuropathologies, including periventricular leukomalacia (PVL), stroke, traumatic injury, multiple sclerosis and dementia [5–7]. Hence, it is important to understand cytodestructive and cytoprotective mechanisms in WM ischemia.

Notably, in addition to ionotropic glutamate receptors (iGluR), glial cells also express metabotropic glutamate receptors (mGluR) widely throughout the brain [8]. mGluR are seven transmembrane domain G protein-coupled receptors (GPCR) and are divided in three groups, based on their second messenger coupling, pharmacology and amino acid sequence [9]. Group I mGluRs (mGluR1 and mGluR5) are positively coupled to phospholipase C (PLC) via Gα_q/11_ and inositol triphosphate (IP_3_) formation, whilst group II (mGluR2 and mGluR3) and group III (mGluR4, mGluR6, mGluR7 and mGluR8) are negatively coupled to adenylyl cyclase (AC) via G_i_/G_0_, and negatively regulate cAMP. Astrocytes prominently express group I mGluR1/5 that elicit a rise in [Ca^2+^]_i_ and can induce their release of glutamate and other neurotransmitters, which can modulate synaptic responses [10, 11]. The role of mGluR in white matter is less well studied, but group I mGluR are protective against ischemia–hypoxia and glutamate-mediated excitotoxity in developing oligodendrocytes [12] and recently we provided evidence that mGluR5 protect astrocytes from ischemic damage in the mouse optic nerve [13]. In vitro, group 1 mGluR5 are highly expressed in oligodendrocyte precursor cells (OPCs) and are down-regulated as they differentiate into mature oligodendrocytes [12, 14]. In vivo studies showed that group I mGluR1/mGluR5 are expressed in immature oligodendrocytes of postnatal white matter and in human postmortem preterm tissue [15, 16], and group II mGluR3 appear to be expressed by OPCs and oligodendrocytes [14, 16]. It is not clear whether mGluR have a cytoprotective role in adult white matter, although as noted above there is considerable evidence that glutamate plays an enduring role in oligodendrocytes and white matter pathology in the adult CNS [4, 6]. To address this, we have compared the effects of mGluR activation on oligodendrocytes in the developing and young adult mouse optic nerve, using the OGD model. Our results provide evidence of a persistent role for mGluR in limiting hypoxic–ischemic oligodendrocyte injury in postnatal and young adult white matter.

## Materials and Methods

### Animals

Mice aged postnatal day (P)8–12 and P30–35 were killed humanely, in accordance with the UK Animals (Scientific Procedures) Act, 1986. Wild type (WT) mice of the C57BL/6 and transgenic mouse strains in which fluorescent reporters are driven by the oligodendroglial genes SOX10-EGFP (gift from William Richardson, UCL, UK) or PLP-DsRed (gift from Frank Kirchhoff, University of Saarland, Germany) were used throughout.

### RT-qPCR

WT mice aged P8–12 and P30–35 were humanely killed and optic nerves and cerebral cortex were rapidly removed. For cortices and postnatal optic nerves, homogenization was performed using a TissueRuptor (Qiagen, Hilden, Germany). For the P30 optic nerve the abundance of myelin interferes with mRNA extraction and the homogenate was passed through a QIAshredder column (Qiagen), then 100 µL chloroform (Sigma) was added and after centrifugation the top aqueous phase containing the RNA was transferred into an Eppendorf, where it was mixed at 1:1 ratio with 70% ethanol. Subsequently, for all samples, RNA extraction was performed using the RNeasy Micro Kit (Qiagen), following the manufacturer’s instructions and total RNA samples were eluted in 10 µl RNA-free water. Genomic DNA was removed using DNase I (Qiagen) and RNA samples were stored at −80 °C prior to use. The purity and concentration of isolated total RNA was assessed using a ND-1000 Spectrophotometer (NanoDrop Technologies, Wilmington, DE, USA), where a ratio of >1.8 indicated high RNA purity. The RT2 First Strand Kit (Qiagen) was used for in vitro transcription of extracted RNA into cDNA, following the manufacturer’s instructions. The quantity of RNA that was transcribed was the same for all samples (500 ng). For RT-qPCR, amplified cDNA was loaded with SYBR green mastermix and DNAase/RNAse free H_2_0 into a custom RT^2^ Profiler™qPCR mGluR 96-well plate array (SABiosciences, Qiagen) for Lightcycler 96 (Roche Diagnostics). Relative gene expression was determined using the ΔΔCt method versus the housekeeping gene Glyceraldehyde-3-phosphate dehydrogenase (GAPDH). Transcript expression data are presented as mean ± standard error of the mean (SEM), and samples compared for significance using ANOVA and post hoc Bonferroni’s tests in Prism v3.02 software (GraphPad).

### Optic Nerve Explant Cultures and Immunocytochemistry

Optic nerve explant cultures were prepared from P8 PLP-DsRed mice as previously described [17]. Briefly, optic nerves were placed directly into 50 µl dissecting medium consisting of high glucose Dulbecco’s Modified Eagle Medium (DMEM) (Sigma-D5671) containing 10% Fetal Calf Serum (FCS; Life Technologies), l-glutamine (Sigma) and 0.1% gentamycin (Life Technologies). Nerves were finely chopped with a scalpel blade and triturated with pipettes of decreasing diameter (P1000 then P200) to further break up the tissue. After adding 50 µl more dissecting medium, the solution was pipetted evenly between poly-l-lysine/laminin coated coverslips (approx. 1 nerve per cover-slip). After 24 h, the dissecting medium was replaced with a low serum (0.5%) modified Bottenstein and Sato (B&S) culture medium [18], without thyroid hormones, in which 10 ng/ml of recombinant human PDGF-a (R&D Systems) was added to encourage OPC proliferation. After 3–4 days in vitro (DIV), the medium was replaced with B&S medium supplemented with 0.5 mM dbc-AMP (Sigma) to promote differentiation of oligodendrocytes, which were identified by their expression of the PLP-DsRed reporter. After 10 DIV, cultures were immersion fixed in 1% paraformaldehyde (PFA) containing 15% picric acid for 10 min. Following washes in phosphate buffered saline (PBS), a blocking stage was performed by incubation in 10% normal goat (NGS) in PBS for 1–2 h, then washes in PBS and incubation overnight with primary antibodies in blocking solution containing 0.01% Triton-X-100: rabbit anti-mGluR5, at 1:1000 (Neuromics); rabbit anti-mGluR2/3, at 1:300 (Upstate). Samples were then washed 3 times in PBS and incubated with the appropriate secondary antibodies conjugated with 488 Alexafluor. Following immunolabelling, coverslips were mounted with Vectashield® (VectorLabs) and imaged using a Zeiss Axiovert LSM710 VIS405 confocal microscope. Controls were performed in which coverslips were preabsorbed with antigen peptide overnight prior to incubation in the primary antibody for mGluR5 and mGluR2/3, and no immunostaining was observed in these controls. Images were acquired using multichannel sequential scanning, narrow bandwidths, and minimal laser power and gain to prevent cross-talk between the channels; a pinhole of 1 airy unit or less was used, with an average of 4 scans per image.

### Oxygen–Glucose Deprivation

Optic nerves from P8–12 or P30–35 transgenic PLP-DsRed or Sox10-EGFP mice were isolated intact and immediately placed in oxygenated *a*CSF at 37 °C for 30 min, comprising (in mM): NaCl,133; KCl, 3; CaCl_2_, 1.5; NaH_2_PO_4_, 1.2; MgCl_2_, 1.0; d-glucose. 10; HEPES, 10; pH 7.3. Controls were incubated for a further 1 h in normal *a*CSF containing 10 mM glucose with 95% O_2_/5% CO_2_. Oxygen–glucose deprivation (OGD) was achieved using the method of Fern and colleagues [19], by incubating nerves for 1 h at 37 °C in glucose-free *a*CSF (osmolarity was maintained by replacing glucose with sucrose), and switching the chamber atmosphere to 95%N_2_/5%CO_2_. The use of 10 mM HEPES buffer ensured the pH remained steady throughout these short experiments and the control data demonstrates that oligodendrocytes remain viable in these conditions. Pharmacological agents were added directly to the *a*CSF solution (purchased from Tocris and used at 100 µM unless otherwise stated): group I/II mGluR agonist ACPD [(±)-1-Aminocyclopentane-*trans*-1,3-dicarboxylic acid]; selective group I mGluR agonist DHPG [(*RS*)-3,5-Dihydroxyphenylglycine]; highly selective group II mGluR agonist LY379268 ((1*R*,4*R*,5*S*,6*R*)-4-amino-2-oxabicyclo[3.1.0]hexane-4,6-dicarboxylic acid); potent protein kinase C (PKC) inhibitor (10µM) Go6976 (5,6,7,13-tetrahydro-13-methyl-5-oxo-12*H*-indolo[2,3-*a*]pyrrolo[3,4-*c*]carbazole-12-propanenitrile); membrane permeable cAMP analogue dbcAMP (N^6^,2′-*O*-dibutyryladenosine 3′,5′-cyclic monophosphate sodium salt, Sigma); membrane permeable activator of adenylyl cyclase forskolin ([3*R*-(3α,4aβ,5β,6β,6aα,10α,10aβ,10bα)]-5-(acetyloxy)-3-ethenyldodecahydro-6,10,10b-trihydroxy-3,4a,7,7,10a-pentamethyl-1*H*-naphtho[2,1-*b*]pyran-1-one, Sigma). At the end of the experiments, nerves were rapidly fixed for 1 h in 4% PFA and whole-mounted with vectashield. Cell counts were performed in whole mounts of fixed optic nerve, using a constant volume of 20 × 20 µm in the *x*-*y*-plane and 15 µm in the *z*-plane, captured using a Zeiss LSM 710 Metaconfocal microscope, as previously described [20]. Images were captured at the midway along the length of the optic nerve, to avoid the cut ends, and at a depth of 15 µm beneath the pial surface. Expression of the transgenic marker EGFP or DsRed identifies viable cells and a decrease in their number reflects the number of cells that died, ruptured and disappeared over the 60 min experimental period. Cell death was also measured in WT mice using propidium iodide (PI, 1 μg/μl; Sigma), which is membrane impermeant in viable cells, from which PI is excluded, and only permeates the membranes of cells that are unviable and will subsequently die; PI staining and EGFP/DsRED imaging was not performed in the same cells. Data were expressed as mean ± SEM, where each ‘*n*’ value represents a separate nerve, and significance was determined by ANOVA and Newman-Keuls multiple comparison post-hoc analysis, using Prism 5.0 (Graphpad).

## Results

### mGluR in the Postnatal and Young Adult Mouse Optic Nerve

**Fig. 1:**
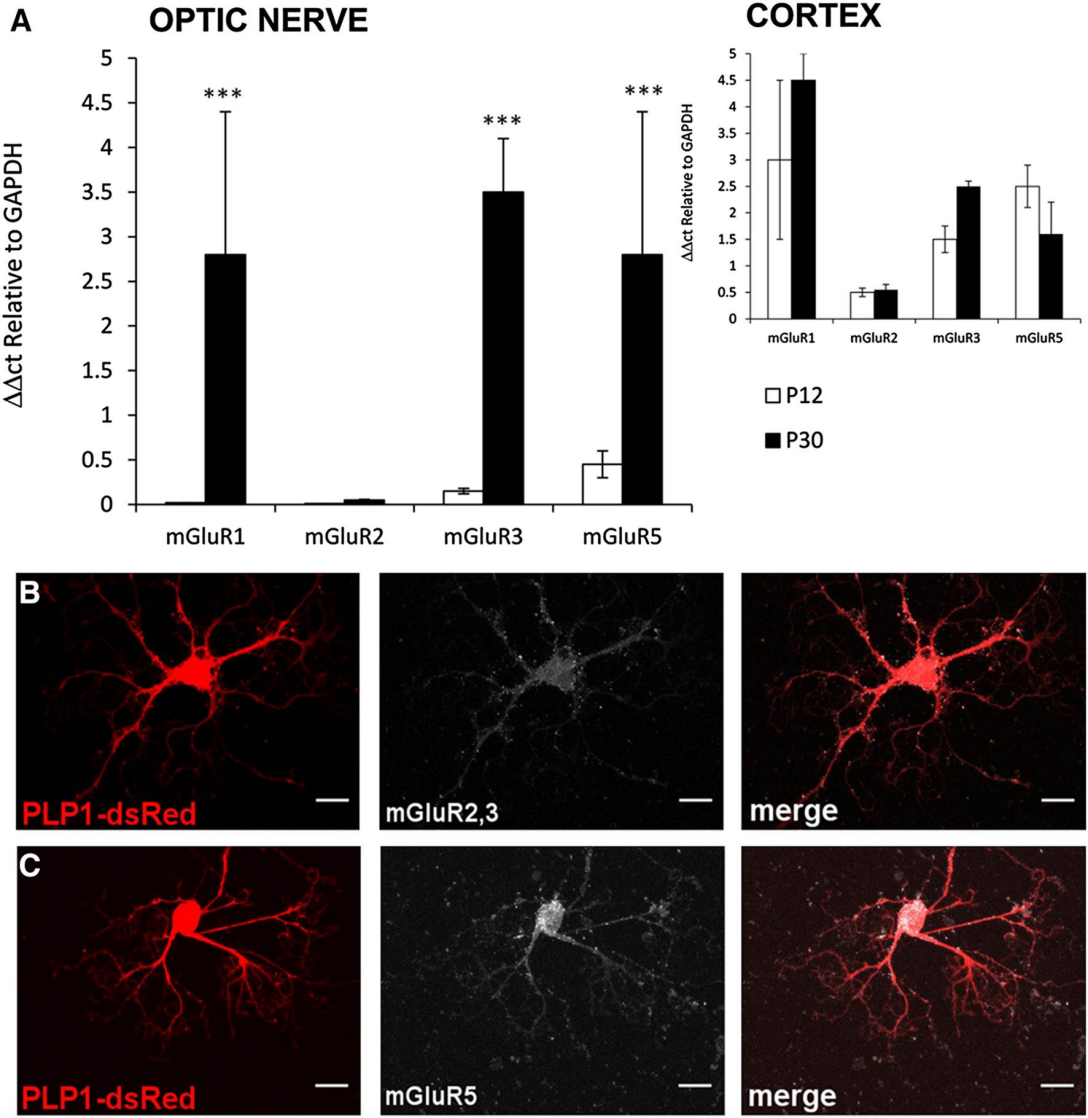
Expression of mGluR in optic nerve oligodendrocytes. **a** RT-qPCR of mGluR subtypes in the postnatal (P8–12) and young adult (P30–35) optic nerve, compared to cortex at the same ages (*inset*); data are expressed as mean ± SEM ΔΔCT relative to GAPDH, ***p < 0.001 determined by ANOVA and post hoc Bonferroni’s test. **b, c** Oligodendrocytes in optic nerve explant cultures from P8 PLP-DsRed reporter mice after 10 DIV were immunolabelled for mGluR2/3 (**d**) and mGluR5 (**e**), illustrating single channels (Di, Dii, Ei, Eii) and the merged channel in which mGluR colocalization with PLP appears white (Diii, Eiii); *scale bars* 10 µm

RT-qPCR was used to analyse expression of group I mGluR (mGluR1 and mGluR5) and group II mGluR (mGluR2 and mGluR3) subtypes in the optic nerve, compared to the cortex, from developing (P8–12) and young adult (P30–35) WT mice, using a custom RT^2^ Profiler™qPCR array (Sabiosciences, Qiagen). In the cortex, transcripts for all subtypes were detected, mGluR1 (Grm1), mGluR3 (Grm3) and mGluR5 (Grm5) being most abundant, and there were no significant differences between developing and young adult (Fig. 1, inset). In contrast, mRNA for mGluR2 was barely detectable at either age in the optic nerve, whereas mGluR1, mGluR3 and mGluR5 were detected and were significantly upregulated between P12 and P30, to levels observed in the cortex (Fig. 1; ANOVA and post hoc Bonferroni’s tests, p < 0.001). The optic nerve does not contain neuronal cell bodies and >95% of mRNA is oligodendroglial and astroglial [19]. The RT-qPCR results indicate a developmental increase in glial mGluR1, mGluR5 and mGluR3. Immunolabelling in optic nerve explant cultures prepared from P8 PLP-DsRed reporter mice confirms that after 10 DIV PLP-DsRed + oligodendrocytes express mGluR5 and mGluR3 (Fig. 1b, c); immunostaining for mGluR5 was greater than for mGluR2/3 and the former appears to be strong in the cell body, which is consistent with the literature [12, 15].

### mGluR Protect Oligodendrocytes from Ischemia–Hypoxia In Situ in the Postnatal Optic Nerve

**Fig. 2:**
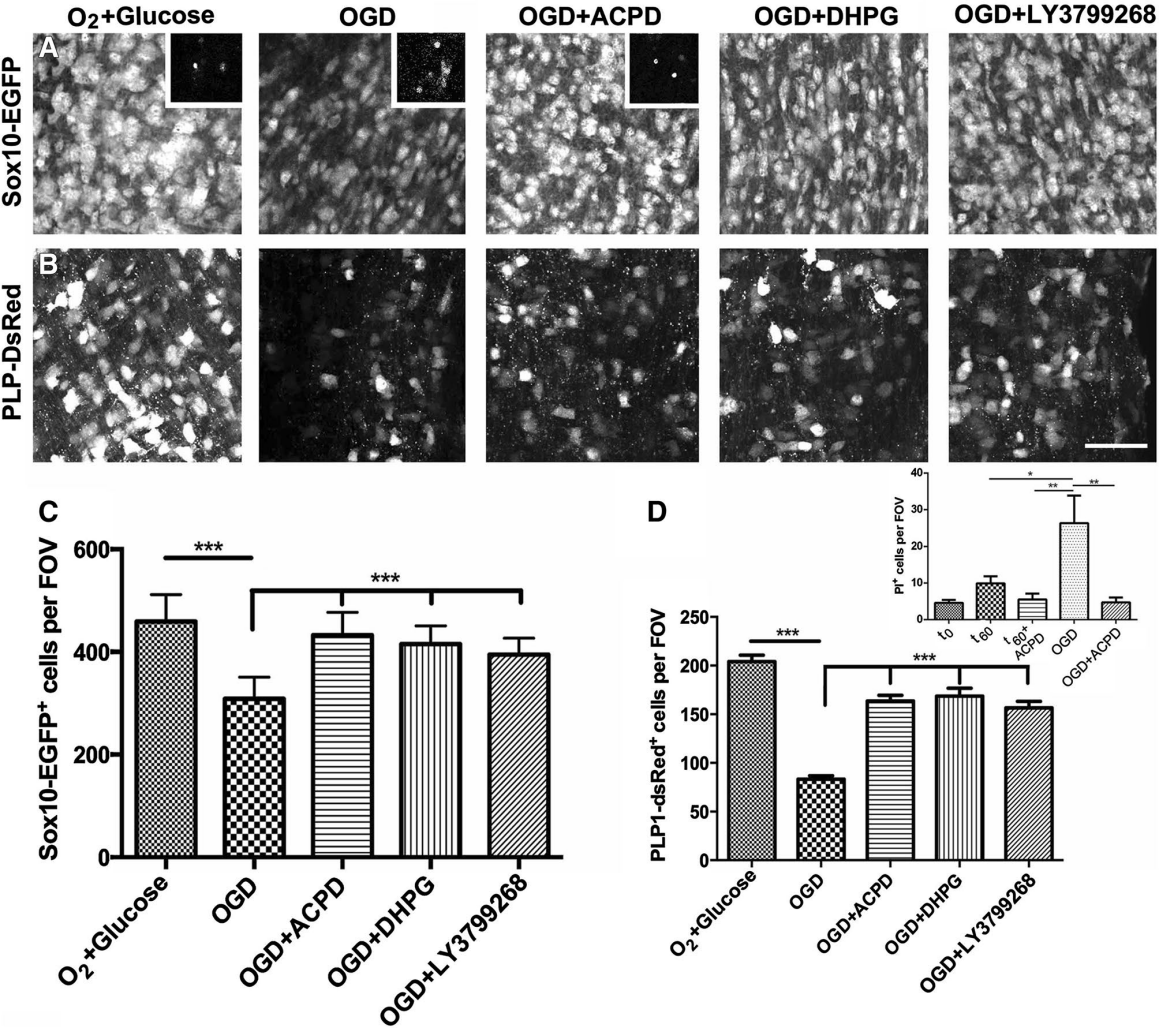
mGluR activation protects postnatal optic nerve oligodendrocytes from OGD. **a, b** Representative confocal images of whole-mounted optic nerves from P8 SOX10-EGFP mice (**a**) and PLP-DsRed mice (**b**) that were exposed to OGD for 1 h, in the presence or absence of the group I/II agonist ACPD, specific group I agonist DHPG, or group II agonist LY379268 (all 100 µM), compared to control nerves incubated in normal *a*CSF with O_2_ + glucose; *scale bar* 50 µm. **c, d** Quantification of the number of SOX10-EGFP^+^ cells (**c**) and PLP-DsRed + cells (**d**), in constant fields of view (FOV) (mean ± SEM, *n* = 6 nerves per experimental group; ***p < 0.001, ANOVA with Newman–Keuls multiple comparison post-hoc analysis). *Insets* in **a** are representative images of PI labeling used to measure cell death and quantification of the number of PI^+^ cells is illustrated in the *inset* in **d**

Immature oligodendrocytes and OPCs are highly sensitive to glutamate-mediated ischemia–hypoxia [6, 21] and mGluR have been shown to protect OPCs from glutamate-mediated excitotoxicity in vitro [12, 22]. The expression of both group I and group II mGluR in PLP + oligodendrocytes shown above suggested they may have a protective role in oligodendrocytes as well as OPCs. Hence, we examined the effects of mGluR activation on oligodendrocytes in situ in the isolated intact mouse optic nerve from mice aged P8–12 (Fig. 2). First, to examine cell death, we used PI labelling in WT nerves; PI is membrane impermeant and excluded from viable cells, and only permeates the membranes of cells that are unviable and will subsequently die. Optic nerves were incubated for 60 min in normoxic or OGD conditions in normal *a*CSF + PI or *a*CSF + PI containing ACPD (Fig. 2a, b, d insets); at the end of the experiments, nerves were rapidly immersion fixed and whole-mounted for examination using a confocal microscope to identify PI labeled dying cells (many cells that died earlier will have lysed and disappeared). Compared to acutely dissected nerves, there was no significant cell death following incubation in normoxic *a*CSF for 1 h, whereas OGD resulted in significant cell death (p < 0.05), compared to normoxic controls, and this was completed blocked by incubation in the general mGluR agonist ACPD, which activates both group I and group II mGluR (insets in Fig. 1a, b, d). These results demonstrate that nerves remain viable in the absence of OGD, as previously reported using the same techniques [19, 20], and supports evidence that mGluR are cytoprotective against OGD in white matter in vivo [15].

To dissect the roles of group I and II mGluR on oligodendrocytes and OPCs specifically, we used transgenic Sox10-EGFP and PLP-DsRed reporter mice (Fig. 2). Sox10 is expressed throughout the oligodendrocyte lineage [23], hence Sox10-EGFP identifies both oligodendrocytes and OPCs (Fig. 2a, c), whereas PLP-DsRed identifies mainly oligodendrocytes (Fig. 2b, d) [24]. Optic nerves were incubated for 60 min in normoxic or OGD conditions in normal *a*CSF or *a*CSF containing one of the mGluR antagonists (Fig. 2) and at the end of the experiments, nerves were rapidly immersion fixed. Nerves were whole-mounted for examination using a confocal microscope and cell counts were performed in each nerve in a constant FOV, comprising 20 × 20 × 15 µm in the *x*-*y*-*z* plane, at a point midway along the length of the optic nerve and 15 µm beneath the pial surface, as previously described [20]. Expression of the transgenic marker EGFP or DsRed identifies viable cells and a decrease in their number reflects the number of cells that have died, ruptured and consequently disappeared over the 60 min experimental period; this avoided the issues of live cell imaging, where tissue swelling and movement results in some cells moving in or out of the confocal plane, while other cells may fade without dying. Compared to normoxic controls, OGD resulted in a significant decrease in oligodendrocytes, in the SOX10-EGFP nerves (Fig. 2a, c, p < 0.001) and PLP-DsRed nerves (Fig. 2b, d, p < 0.001). Incubation with ACPD, the specific group I agonist DHPG, or group II agonist LY379268 both had a significant cytoprotective effect against OGD in Sox10-EGFP + oligodendrocytes (Fig. 2a, c, p < 0.001) and PLP-DsRed + oligodendrocytes (Fig. 2b, d, p < 0.001). The total number of oligodendrocytes that died during the 60 min experimental period is measured by the decrease in the total number of cells expressing the transgenic markers (Fig. 2c, d) and is greater than the number of PI stained cells per FOV at the end of the experiment (Fig. 2d, inset), which is a measure of the number cells undergoing cell death at that time. The results indicate mGluR have a cytoprotective effect on oligodendrocytes in the postnatal optic nerve, in support of in vitro studies [12, 22].

### Group I mGlu, Protect Oligodendrocytes from Ischemia–Hypoxia in Young Adult Optic Nerve

**Fig. 3:**
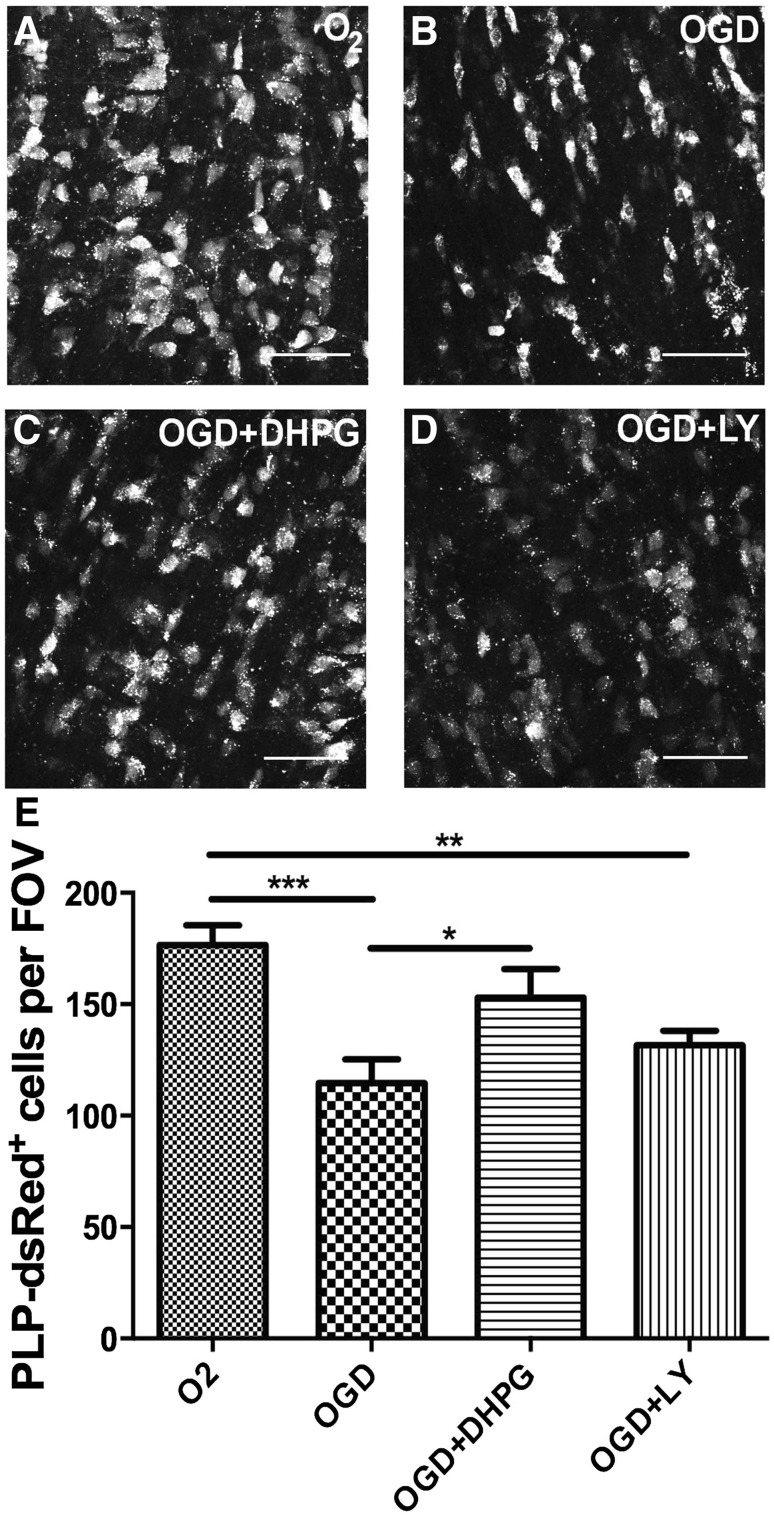
mGluR activation protects oligodendrocytes in young adult optic nerves from OGD. Optic nerves from P33 PLP1-DsRed reporter mice were exposed to 1 h OGD in the presence or absence of the specific group I agonist DHPG or group II agonist LY379268 (both at 100 µM), compared to control nerves incubated in normal *a*CSF with O_2_ + glucose. **a–d** Representative images of PLP1-DsRed^+^ oligodendrocytes in isolated intact optic nerves incubated in O_2_ (**a**), OGD (**b**), OGD + DHPG (**c**), or OGD + LY (**d**); *scale bars* 50 µm. **e** Quantification of the number of PLP1-DsRed ^+^ cells in constant fields of view (FOV) (mean ± SEM, *n* = 6 nerves per experimental group; *p < 0.05, **p < 0.01, ***p < 0.001, ANOVA with Newman–Keuls multiple comparison post-hoc analysis)

Mature oligodendrocytes are susceptible to glutamate-mediated excitotoxcity in adult white matter pathology, including ischemia–hypoxia and multiple sclerosis [6, 7], and increased expression of group I and group II mGluR suggests their cytoprotective role may persist in the adult optic nerve. Hence, we examined the effects of mGluR activation on oligodendrocytes in situ in the isolated intact mouse optic nerve from mice aged P33 (Fig. 3). After 1 h OGD in the young adult nerve, there was a significant 30% loss of PLP1-DsRed^+^ oligodendrocytes compared to normoxic controls (Fig. 2a, b, e, p < 0.001). Incubation with the group I mGluR agonist DHPG significantly protected oligodendrocytes from OGD (Fig. 3b, c, e, p < 0.05), and there was no significant difference between OGD + DHPG and normoxic controls (Fig. 3a, c, e, p > 0.05). In contrast, the group II mGluR agonist LY379268 did not protect against OGD (Fig. 3d, e); there was no significant difference between OGD and OGD + LY379268 (Fig. 3b, d, e, p > 0.05), and the number of oligodendrocytes was significantly less than in OGD + DHPG and normoxic controls (Fig. 3a, d, e, p < 0.01). The results demonstrate that group I mGluR are cytoprotective against ischemia–hypoxia in oligodendrocytes in the young adult optic nerve in situ, but the cytoprotective effects of group II mGluR declines with development.

### The Protective Effect of Group I mGluR on Oligodendrocytes is Mediated by PKC

**Fig. 4:**
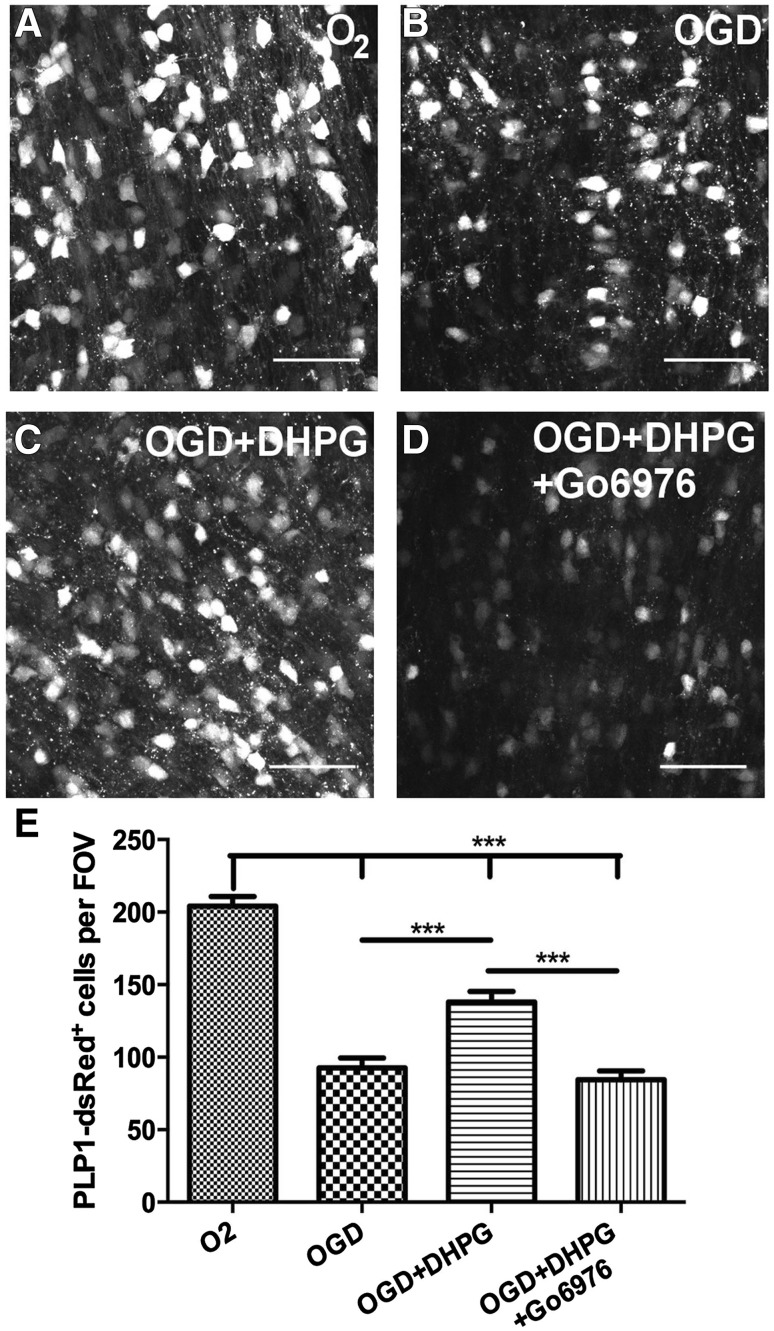
Protective effect of group I mGluR in oligodendrocytes depends on PKC activation. Optic nerves from P11 PLP1-DRed reporter mice were exposed to 1 h OGD in the presence of the group I mGluR agonist DHPG (100 µM) and presence or absence of the PKC inhibitor Go6976 (10 µM), compared to normoxic controls incubated in normal *a*CSF with O_2_ + glucose. **a–d** Representative images of PLP1-DsRed^+^ oligodendrocytes in isolated intact optic nerves incubated in O_2_ (**a**), OGD (**b**), OGD + DHPG (**c**), or OGD + DHPG + Go6976 (**d)**; scale bars = 50 µm. **e** Quantification of the number of PLP1-DsRed ^+^ cells in constant fields of view (FOV) (mean ± SEM, *n* = 5 nerves per experimental group; ***p < 0.001, ANOVA with Newman–Keuls multiple comparison post-hoc analysis)

Group I mGluR act via phosholipase C (PLC) and an IP_3_-dependent increase of intracellular Ca^2+^ to activate protein kinase C (PKC), which mediates the protective effect of mGluR in hypoxic-ischemic injury in OPCs in vitro [12]. To examine this in oligodendrocytes, the PKC inhibitor Go6976 (10 µM) was used in conjunction with the group I mGluR agonist DHPG, in optic nerves from P11 PLP1-DsRed reporter mice (Fig. 4). The cytoprotective effect of DHPG was completely abolished by Go6976, with the number of PLP + oligodendrocytes in OGD + DHPG + Go6976 being not significantly different than in OGD alone (Fig. 4b, d, p < 0.001), and significantly less than in OGD + DHPG (Fig. 4c, d, e, p < 0.001), or normoxic controls (Fig. 4a, d, e, p < 0.001). The results demonstrate that group I mGluR mediate their pro-survival effect on PLP + oligodendrocytes via PKC activation.

### The Protective Effect of Group II mGluR on Immature Oligodendrocytes is Dependent on cAMP

**Fig. 5:**
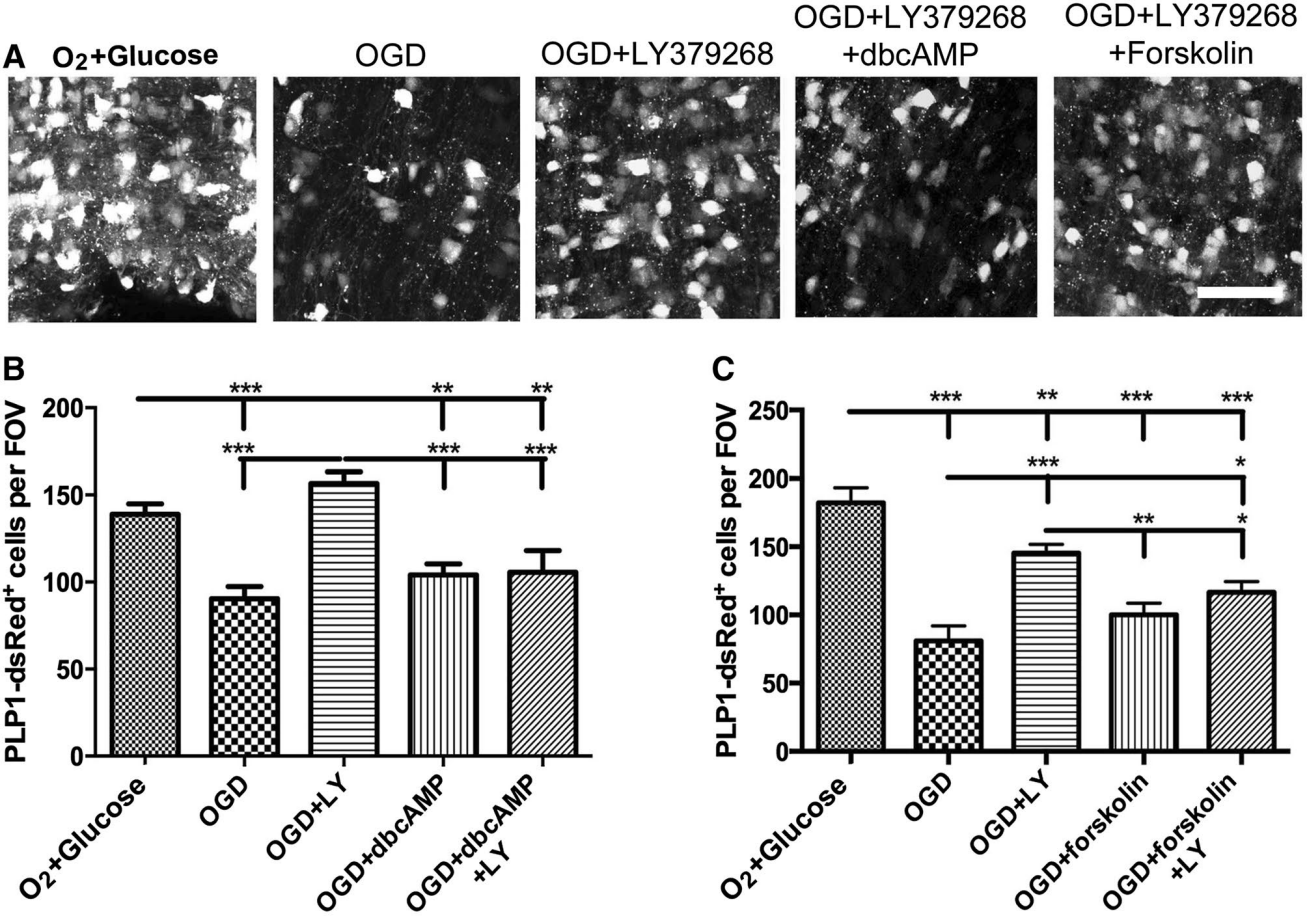
Protective effect of group II mGluR in oligodendrocytes depends on inhibition of adenylyl cyclase and reduced intracellular cAMP. Optic nerves from P11 PLP1-DsRed reporter mice were exposed to 1 h OGD in the presence or absence of the group II mGluR agonist LY379268 (100 µM), together with the membrane permeable cAMP analogue dbcAMP (100 µM) or forskolin (100 µM) to activate adenylyl cyclase and raise endogenous cAMP. **a** Representative images of PLP1-DsRed^+^ oligodendrocytes in isolated intact optic nerves in normoxic controls (O_2_ + Glusose), OGD, OGD + LY379268, OGD + LY379268 + dbcAMP and OGD + LY379268OGD + forskolin; *scale bar* 50 µm. **b, c** Quantification of the number of PLP1-DsRed ^+^ cells in constant fields of view (FOV) to determine the effects of dbcAMP (**b**) and forskolin (**c**) on the protective effects of LY379268 (mean ± SEM, *n* = 5 nerves per experimental group; *p < 0.05, **p < 0.01, ***p < 0.001, ANOVA with Newman–Keuls multiple comparison post-hoc analysis)

The results presented above demonstrated that group II mGluR protect immature oligodendrocytes against OGD in postnatal nerves. Since mGluR2 were barely detectable in the postnatal nerve, these effects are most likely mediated by mGluR3, which have an established cytoprotective role in neurons and astrocytes by inhibition of adenylyl cyclase (AC) and reduction of cAMP levels [25, 26]. We examined this in optic nerves from P11 PLP-DsRed mice, using the membrane permeable cAMP analogue dbcAMP (100 µM) to exogenously raise intracellular cAMP and the AC activator forskolin (100 µM) to raise endogenous cAMP. Both dbcAMP and forskolin counteracted the cytoprotective actions of group II mGluR activation in oligodendrocytes (Fig. 5). Incubation with dbcAMP had no effect on OGD-mediated oligodendrocyte loss, but the number of PLP-DsRed + oligodendrocytes was significantly less in OGD + dbcAMP + LY379268 than in OGD + LY379268 (p < 0.001), or normoxic controls (p < 0.01) (Fig. 5a, b). Similarly, incubation in forskolin, to activate AC and raise endogenous cAMP, had no effect on oligodendrocyte loss in OGD, but the number of PLP-DsRed + oligodendrocytes was significantly less in OGD + forskolin + LY379268 than in OGD + LY379268 (p < 0.05), or normoxic controls (p < 0.001) (Fig. 5a, c). The results demonstrate that the cytoprotective effect of group II mGluR on oligodendrocytes is dependent on a decrease in cAMP, which can be reversed by pharmacologically raising cytoplasmic cAMP.

## Discussion

Oligodendrocytes are highly susceptible to glutamate-mediated excitoxicity in the developing and adult brain [6]. Here, we show that mGluR protect oligodendrocytes from ischemic-hypoxic injury in situ in the optic nerve of postnatal and young adult mice. Group I and II mGluR are developmentally upregulated in the mouse optic nerve, indicating an enduring role for mGluR in glial glutamate signaling, which is a prominent feature of white matter [7]. Group I mGluR are cytoprotective for oligodendrocytes at all stages of differentiation and development, whereas the cytoprotective effect of group II mGluR was lost in the P30 nerve. The results suggest that targeting specific mGluRs at different developmental ages could contribute to strategies for protecting immature and mature oligodendrocytes in neuropathologies involving excitoxicity and ischemia–hypoxia.

In vivo expression of mGluR in the optic nerve was confirmed by RT-qPCR, which identified mRNA for mGluR1, mGluR3 and mGluR5 in the postnatal optic nerve, and these were markedly upregulated at P30, to levels observed in the cortex. The bulk of mRNA in the optic nerve is from oligodendrocytes and astrocytes [19], and our previous studies have demonstrated functional mGluR in optic nerve glia [27]. Our results support evidence that oligodendrocytes express group I mGluR1/mGluR5 in postnatal rodent white matter and in human postmortem preterm tissue [15, 16], and our finding that group II mGluR3 are expressed by PLP + oligodendrocytes from postnatal nerves is consistent with in vitro studies [14, 16].

In the postnatal optic nerve, OGD-resulted in a 70% loss of PLP + oligodendrocytes compared to 30% in the P30 nerve. The postnatal mouse optic nerve correlates developmentally to human fetal white matter that is highly susceptible to ischemia–hypoxia, resulting in hypomyelination in periventricular leukomalacia (PVL) and cerebral palsy [28]. Our results support evidence that immature oligodendrocytes are more vulnerable than mature oligodendrocytes to ischemia–hypoxia [29], but also support evidence that CNS white matter is vulnerable to ischemic injury in older animals [4]. Interestingly, a key aspect of glial and axonal demise in ischemic white matter is the massive release of glutamate into the extracellular space [3]. The cytodestructive effects of glutamate in oligodendrocytes and myelin are mediated by AMPA- and NMDA-type iGluR and blockade of NMDA receptors is protective for oligodendrocytes and axonal integrity [2, 5, 19, 30, 31]. Our results indicate that mGluR serve to potentially protect oligodendrocytes, but the loss of oligodendrocytes in ischemia–hypoxia indicates that without pharmacological intervention the cytodestructive effects of iGluR outweigh the cytoprotective effects of mGluR.

Group I and II mGluR were both protective for oligodendrocytes in the postnatal optic nerve, whereas only group I were protective in young adults, and were less effective than in postnatal nerves. The cytoprotective effect mediated by group I mGluR requires activation of PKC, which has also been shown in vitro in OPCs subjected to kainate exposure and OGD [12]. The protective effects of group II mGluR in immature oligodendrocytes support studies showing LY379268 is neuroprotective in ischemia [25]. The protective effect of LY379268 in the optic nerve is most likely mediated by mGluR3, since mGluR2 were barely detectable, and is dependent on inhibition of adenylyl cyclase and reduced cAMP levels, which in astrocytes has been shown to result in activation of the prosurvival PI3K/Akt pathway [26].

Ischemia–hypoxia causes depolarization of oligodendrocytes and a consequent rise in [Na^+^]_i_, which result in reversal of the Na^+^–Ca^2+^-exchanger (NCX) and Ca^2+^ influx, triggering apoptosis [32, 33]. Notably, activation of group I mGluR has been reported to activate NCX in neurons [34, 35], which may be involved in the protective effects of mGluR5 in the optic nerve. Another important factor in ischemia–hypoxia is decreased astroglial glutamate uptake, which results in raised extracellular glutamate and is a major cause of oligodendrocyte loss in OGD [6]. mGluR5 are cytoprotective for astrocytes [13, 36] and stimulate astroglial glutamate uptake [37], which could have indirect protective effects on oligodendrocytes by attenuating the loss of astroglial homeostatic functions in the optic nerve. Furthermore, mGluR can stimulate release of brain derived neurotrophic factor (BDNF) from astrocytes, which is neuroprotective and decreases myelin loss following demyelination [38]. mGluR also increase BDNF release from oligodendrocytes, through a PLC pathway [39]. In addition, group I mGluR inhibit microglial production of NO and reactive oxygen species, which are cytotoxic for oligodendrocytes [40]. It is likely that mGluR activation activates multiple mechanisms in the optic nerve that are cytoprotective for oligodendrocytes.

The results provide novel evidence that group I and group II mGluR protect oligodendrocytes from ischemia–hypoxia. The apparent developmental decrease in the efficacy of mGluR-mediated cytoprotection contrasts with the marked upregulation of mGluR1/5 and mGluR3 in the adult optic nerve, suggesting an important unresolved function for mGluR in adult white matter. Overall, this study indicates that mGluR represent a potential therapeutic strategy to protect oligodendrocytes in pathologies that involve hypoxia–ischemia and excitoxicity, which is relevant to stroke, PVL and cerebral palsy, multiple sclerosis, neurodegenerative diseases and dementia.
